# The impact of occupational exposures on infection rates during the COVID-19 pandemic: a test-negative design study with register data of 207 034 Dutch workers

**DOI:** 10.5271/sjweh.4086

**Published:** 2023-05-01

**Authors:** Iris Eekhout, Martie van Tongeren, Neil Pearce, Karen M Oude Hengel

**Affiliations:** 1 Netherlands Organisation for Applied Scientific Research TNO, Unit Healthy Living, Leiden, The Netherlands.; 2 Centre for Occupational and Environmental Health, Centre for Epidemiology, Division of Population Health, Health Services Research and Primary Care, School of Health Sciences, Faculty of Biology, Medicine and Health, The University of Manchester, Manchester Academic Health Science Centre, Manchester, United Kingdom.; 3 London School of Hygiene & Tropical Medicine, London, United Kingdom.

**Keywords:** job exposure matrix, mitigating measure, precarious work, SARS-CoV-2, transmission risk

## Abstract

**Objective:**

This study aimed to investigate the effects of occupational exposures on the risk of a positive COVID-19 test, and whether this differed across pandemic waves.

**Methods:**

Data from 207 034 workers from The Netherlands with test data on COVID-19 from June 2020 until August 2021 were available. Occupational exposure was estimated by using the eight dimensions of a COVID-19 job exposure matrix (JEM). Personal characteristics, household composition and residence area were derived from Statistics Netherlands. A test-negative design was applied in which the risk of a positive test was analyzed in a conditional logit model.

**Results:**

All eight dimensions of occupational exposure included in the JEM increased the odds of a positive test for the entire study period and three pandemic waves [OR ranging from 1.09, (95% confidence interval (CI) 1.02–1.17) to 1.77 (95% CI 1.61–1.96)]. Adjusting for a previous positive test and other covariates strongly reduced the odds to be infected, but most dimensions remained at elevated risk. Fully adjusted models showed that contaminated work spaces and face covering were mostly relevant in the first two pandemic waves, whereas income insecurity showed higher odds in the third wave. Several occupations have a higher predicted value for a positive COVID-19 test, with variation over time.

**Discussion:**

Occupational exposures are associated with a higher risk of a positive test, but variations over time exist in occupations with the highest risks. These findings provide insights for interventions among workers for future pandemic waves of COVID-19 or other respiratory epidemics.

Since the first discovery of the severe acute respiratory syndrome coronavirus 2 (SARS-CoV-2) at the end of 2019 (1), the incidence of COVID-19 rapidly increased with a substantial number of infected people across all countries around the world. Direct from the beginning of the pandemic, governments implemented far-reaching measures to change population behaviors in order to limit social contacts outside the household, and large scale testing for infections (2). This was extended with mass vaccination programs, starting with healthcare workers in January 2021 followed by other workers from February onwards in The Netherlands.

Even though the pandemic had a high impact on the entire working population, the risk of exposure to Sars-Cov-2 at work differed across occupations. During the strict lockdown in the first wave, exposure to Sars-Cov-2 was highest in essential occupations, as these occupations demand on-site work and involve close proximity with colleagues or the general public without the availability of personal protective equipment (3). Examples of occupations at increased risk of infection or death from COVID-19 were healthcare workers (4–11), workers in the transport sector (4, 7, 8, 11–13) and cleaners (5, 9). The risk of exposure to Sars-Cov-2 differed not only across occupations but also across pandemic waves. Whereas health care workers had the highest infection rates during the first wave (14), other workers were at higher risk (eg, cooks, bartenders) in later phases of the pandemic due to reopening of sectors while having less access or usage of face coverings and other preventive measures (10, 11).

Even though previous studies have shown that the workplace appears to be one of the key-settings for Sars-Cov-2 exposure, the most important setting in the spread of Sars-Cov-2 remained the household (15). The transmission within households even increased over time due to relaxation of restrictions (16). Also, an increase in transmissibility was noticed between children and adults after reopening of schools and because children became more susceptible to newer variants such as Delta and Omicron (17, 18). Outside the household, living in urban areas with higher population density and increased use of public transport is also a potential risk factor for the spread of Sars-Cov-2 (19, 20). Accordingly, the extent to which occupational exposures are associated with a COVID-19 infection, other settings and factors, such as household composition and living areas, should also be considered.

As the COVID-19 pandemic progressed, the testing facilities and guidelines changed in The Netherlands and testing increased both for people with and without symptoms. From June 2020 onwards polymerase chain reaction (PCR) tests and rapid antigen COVID-19 tests became widely available in The Netherlands. Widespread testing enables researchers to obtain a better understanding of risk factors for infections in the COVID-19 pandemic by using a test-negative design. In this design, people who are tested positive are defined as ‘cases’, while others – symptomatic or asymptomatic – who are tested negative are ‘controls’ (21). The test-negative design can be used to detect differences in risk factors between symptomatic persons who have COVID-19 (test-positives) and those who have other respiratory infections or no infection at all (test-negatives) (22). This design has quickly gained popularity recently to study risk infections or effect of vaccinations because it attempts to reduce confounding bias due to health-care-seeking behavior and severity of the symptoms (22). Direct comparisons of test-positives to test-negatives by applying this design might be helpful to gain insight into specific risk factors for becoming infected and symptomatic, and to unravel the effects of occupational exposure during the pandemic.

The aim of the current study was to systematically quantify the associations between occupational exposure and the risk of a positive test during the pandemic, while correcting for previous positive test result(s), personal factors, household composition and residence area for the entire pandemic period and across three different pandemic waves by applying the test-negative design.

## Methods

### Data

Different data sources from Statistics Netherlands and Municipal Health Services were used which are linked by a unique personal identification number. Firstly, the Dutch Labour Force Survey (DLFS) is an annual rotating panel in which a representative group of people aged ≥15 years who live in The Netherlands received five questionnaires over a period of 12 months on a variety of topics, including sociodemographic factors and occupation (23). People participating in the DLFS over the period 2010–2021 were included in the current study.

Secondly, the personal records database was added to retrieve data on education and household composition.

Thirdly, information regarding whether and when a participant died was also included.

Fourthly, DLFS data were enriched with the social statistical database (SSB) over the same period (24). This dataset contains monthly objective information on main income components, social benefit pensions and gross wages derived from the Dutch tax.

Fifthly, data on the residence address was used to define the province of residence and urbanity of residence area.

Lastly, these data from Statistics Netherlands were matched with the CoronIT database from the Municipal Health Services in The Netherlands which consist of Sars-Cov-2 test results for the period 1 June 2020 to 31 August 2021. In this period, testing was available for everyone experiencing symptoms compatible with COVID-19 as well as for contact tracing. This database includes all test results from residents in The Netherlands who applied for a test at the Municipal Health Services, but does not contain test data from hospitals and commercial test centers.

### Study population

Participants in paid employment and with information on their job title were selected from the DLFS (N=1 356 982). Participants were only included when they were aged 18–74 years and still employed in June 2020 (ie, working population). Data from these participants were linked to the personal records database (N=1 279 943). Only participants who were tested for COVID-19 during the entire study period were included (N=616 855). Based on the SSB, individuals were also excluded if they changed their job or employment status (ie, self-employment, receiving benefits) between their participation in the DLFS and June 2020 (N=409 821). The final sample consisted of 207 034 workers. When the participant reached the age of 75 years old, changed jobs, or left paid employment during the test data period, the test results were excluded from that specific time point onwards.

### COVID-19 test data

The primary outcome was the binary serological status (positive versus negative) for SARS-CoV-2—a marker of natural infection systematically recorded by the Municipal Health Services and retrieved from the CoronaIT dataset. The database consists of information on test date and test result for each test at individual level in the period 1 June 2020 to 31 August 2021. In this period, the Alpha variant was dominant up to June 2021 followed by the Delta variant in July and August 2021. We removed any positive tests following within 8 weeks of the first positive test. A positive test result at a previous time was included in the analyses as covariate. A previous positive test results was related with a lower risk to be infected again, but is not equally distributed across jobs. It was not possible to differentiate between the two tests (PCR and rapid antigen test) within the database.

### Occupation

Self-reported on occupation were retrieved from the DFLS and coded by Statistics Netherlands into four-digit job codes according to the International Standard Classification of Occupations 2008 (ISCO-08) (25).

### Occupational exposure to Sars-Cov-2

Exposure to Sars-Cov-2 at work at level of occupation was determined by an expert COVID-19 job exposure matrix (COVID-19-JEM; 26) The COVID-19-JEM exists of eight dimensions: four dimensions on transmission risk (number of contacts, nature of contacts, contaminated workspaces (eg, sharing surfaces, tools, and equipment) and location); two dimensions on mitigation measures (social distance and face covering); and two dimensions indicating precarious work (proportion of workers per occupation with income insecurity and proportion of migrant workers). The COVID-19-JEM was developed as a basic state of the pandemic, meaning that general measures are taken (social distancing, washing hands, face covering in public places, working from home as possible) but without closure of sectors. For each dimension, all 436 occupations within the ISCO-08 were assigned an exposure risk ranging from 0–3 (no, low, intermediate, and high probability). For the current study, the Dutch version of the COVID-19-JEM was used, which has been described extensively elsewhere (26).

### Other variables

Age, gender, educational level, ethnic background, household position, having children living at home, province of the residence area and the urbanity of the residence area were also included. For each variable, information of the participant was set at time of test date.

Age, ethnicity and gender were retrieved from the DFLS. Ethnic background was defined as the country of origin and categorized into a Dutch background, a Western background and a non-Western background. Education was based on the personal records database and defined as the highest level of education completed, and categorized into low, intermediate and high.

Household position is obtained from the personal records database and categorized into four categories: single household, single parent, part of a couple, and other. Having children living at home was also included as variable. Children living at home was categorized into (i) having children until the age of 12 years (ii), having children of ≥12 years or older (iii), having children both < and ≥12 years of age, and (iv) not having children living at home.

Province and urbanity of residence area were based on the personal records database. Province of residence area consisted of the 12 provinces in The Netherlands. The urbanity of the residence area was defined as the number of addresses per square kilometer and was categorized as non-urban (<500), mildly (≥500 and <1000), moderately (≥1000 and <1500), highly (≥1500 and <2500), and very highly (≥2500).

### Statistical analyses

A test-negative design was applied in which the risk for a positive test was analyzed in a conditional logit model with test date as strata, controlling for age, gender and previous positive test result. This is essentially a case–control type of analysis – where cases test positive and controls test negative – intended to remove or minimize selection biases that can arise when some subgroups of the population get tested more than others (27). As a first step, minimally adjusted models were estimated for each JEM dimension and the overall JEM score as well as for each potential confounder. Secondly, these minimally adjusted models for the JEM dimensions were each additionally adjusted for all other potential confounders (ie, previous positive test, educational level, ethnical background, household position, children living at home, province and urbanicity). Thirdly, all JEM dimensions were modeled with the same confounder adjustment. For the full multiple regression model, the predicted values were extracted per occupation at the 3-digit ISCO 2008 aggregate level. The conditional logit models were estimated with the “clogit” function from survival package using the Efron approximation method to estimate the likelihoods (28) in R statistical software version 4.1.3 (29).

All analyses were conducted for the overall test period and for each wave separately. We defined three different time periods based on the lowest infection rates in The Netherlands (30) – wave 1: 1 June 2020 to 8 February 2021; wave 2: 9 February to 28 June 2021; and wave 3: 29 June to 31 August 2021.

## Results

The final study population consisted of 207 034 workers with a mean age of 45.2 [standard deviation (SD) 12.7] years, and half of them were male (49%; table 1). Of all participants, 12% was low educated, 42% was intermediate educated, and 46% was higher educated. The majority are of Dutch origin (86%), live in household as part of a couple (76%) and have children living at home (62%). Just over half the participants live in areas with high or very high population densities (53%). The risk to be exposed to Sars-Cov-2 at work, as defined by the eight dimensions of the COVID-19-JEM, can be found in the supplementary material (www.sjweh.fi/article/4086), table S1. The characteristics of the study population are in general similar over all pandemic waves. Over the entire study period, 9% of all tests were positive, and the percentage was slightly higher in the first wave (ie, 10%) as compared to waves 2 and 3 (both 9%). Of all workers, 19% of the people had at least one positive test (wave 1: 15%, wave 2: 13% and wave 3:10%).

**Table 1:** Characteristics and positive test rates of the study population (N=207 034) for the entire study period and for each pandemic wave ^a^ [SD=standard deviation]

		Total (N=207 034)				Wave 1 (N=147 969)				Wave 2 (N=106 082)				Wave 3 (N=34 601)		
		%	N	Mean (SD)		%	N	Mean (SD)		%	N	Mean (SD)		%	N	Mean (SD)
Demographics																
	Age			45.17 (12.67)				44.71 (12.58)				45.09 (12.44)				43.22 (12.99)
	Gender (male)	49.3				47.8				47.9				47.0		
Educational level																
	Low	12.4				11.7				11.5				10.1		
	Intermediate	41.6				40.9				40.3				40.0		
	High	46.0				47.5				48.2				49.9		
Ethnic background																
	Dutch	86.3				86.5				86.7				84.6		
	Non-Western	6.5				6.4				6.2				8.1		
	Western	7.1				7.0				7.1				7.4		
Household position																
	Single household	11.6				11.6				11.0				12.4		
	Single parent	4.7				4.9				4.7				5.0		
	Part of a couple	75.9				76.0				77.3				72.5		
	Other	7.8				7.6				7.0				10.0		
Children living at home																
	None	38.1				37.7				36.6				35.8		
	Children < 12 years	22.0				23.8				25.8				22.3		
	Children ≥ 12 years	33.0				31.6				30.7				35.4		
	Children in both age groups	6.9				6.9				6.9				6.5		
Residence area																
Province																
	Groningen	3.0				3.0				3.1				3.5		
	Drenthe	2.9				2.8				2.9				2.7		
	Flevoland	2.1				2.0				2.0				2.1		
	Friesland	3.4				3.2				3.4				3.1		
	Gelderland	12.8				12.8				12.6				13		
	Limburg	7.1				7.2				7.0				6.1		
	Noord-Brabant	16.6				16.3				17.1				16.4		
	Noord-Holland	14.8				14.9				14.6				15.5		
	Overijssel	7.0				6.9				6.7				7.2		
	Utrecht	8.4				8.6				8.3				9.1		
	Zeeland	2.0				1.9				2.1				2.0		
	Zuid-Holland	20.1				20.3				20.2				19.4		
Urbanity of residence area																
	Non-urban	7.4				7.2				7.4				6.7		
	Mildly	23.3				22.9				23.3				21.4		
	Moderate	16.2				16.3				15.8				15.5		
	High	31.4				31.4				31.4				31.8		
	Very High	21.8				22.2				22.0				24.7		
Positive test rates																
	Number of tests		417 509				220 333				156 864				40 312	
	Positive tests	9.4	39 278			10.0	21 936			8.8	13 810			8.8	3532	
	People with positive tests	18.8	38 824			14.8	2185			12.9	13 775			10.2	3532	
Average tests per person				1.14 (1.09)				1.14 (1.09)				1.17 (1.09)				1.18 (1.09)

^a^ Wave 1: June 2020 to February 8 2021, Wave 2: February 9 to June 28 2021, Wave 3 June 29 to August 31 2021.

Table 2 shows the results of minimally adjusted analyses, showing odds ratios (OR) for a positive test for all factors included in the analyses. The results of these analyses show that for all dimensions of occupational exposure increased odds of a positive test were observed for the higher risk categories compared to no risk category. The only exception was for the high risk category for the nature of contacts during wave 2.

**Table 2:** Minimally adjusted odds ratios ^a^ for occupational exposures and other factors with a positive COVID-19 test for the entire study period and for each pandemic wave ^b^ **Bold indicates statistical significant (P<0.05)**.

		Total OR (95% CI)		Wave 1 OR (95% CI)		Wave 2 OR (95% CI)		Wave 3 OR (95% CI)
Previous positive test (ref: No)		**0.41 (0.37–0.45)**		**0.53 (0.43–0.66)**		**0.40 (0.35–0.45)**		**0.35 (0.29–0.43)**
Demographics								
	Age	**1.01 (1.01–1.01)**		**1.01 (1.01–1.01)**		**1.01 (1.01–1.01)**		**0.97 (0.97–0.98)**
	Gender (ref: female)	**1.29 (1.26–1.31)**		**1.27 (1.24–1.31)**		**1.30 (1.26–1.34)**		**1.26 (1.18–1.35)**
Educational level								
	Low	**1.84 (1.79–1.90)**		**1.73 (1.66–1.80)**		**1.98 (1.88–2.08)**		**1.71 (1.54–1.91)**
	Intermediate	**1.51 (1.48–1.54)**		**1.45 (1.41–1.49)**		**1.56 (1.50–1.62)**		**1.56 (1.45–1.68)**
	High	ref		ref		ref		ref
Ethnic background								
	Dutch	ref		ref		ref		ref
	Non-Western	**1.60 (1.55–1.66)**		**1.75 (1.67–1.83)**		**1.48 (1.39–1.57)**		**1.38 (1.24–1.54)**
	Western	0.97 (0.93–1.01)		0.95 (0.90–1.01)		0.97 (0.91–1.04)		1.09 (0.96–1.23)
Household position								
	Single household	ref		ref		ref		ref
	Single parent	**1.14 (1.08–1.21)**		**1.17 (1.08–1.26)**		**1.17 (1.06–1.29)**		**1.11 (0.92–1.33)**
	Part of a couple	**1.18 (1.14–1.23)**		**1.30 (1.24–1.36)**		**1.15 (1.09–1.22)**		**0.86 (0.77–0.95)**
	Other	**2.14 (2.04–2.25)**		**2.12 (1.99–2.27)**		**2.11 (1.94–2.29)**		**1.74 (1.53–1.96)**
Children living at home								
	None	ref		ref		ref		ref
	Children <12 years	**0.74 (0.72–0.76)**		**0.78 (0.75–0.81)**		**0.77 (0.73–0.81)**		**0.60 (0.54–0.67)**
	Children ≥ 12 years	**1.45 (1.42–1.49)**		**1.46 (1.41–1.50)**		**1.50 (1.44–1.56)**		**1.37 (1.27–1.48)**
	Children in both age groups	**1.29 (1.24–1.35)**		**1.38 (1.31–1.45)**		**1.25 (1.17–1.34)**		**1.24 (1.08–1.42)**
Occupational exposure								
Number of contact								
	Working at home/alone	ref		ref		ref		ref
	<10 workers/day	**1.28 (1.24–1.31)**		**1.24 (1.19–1.28)**		**1.33 (1.27–1.39)**		**1.24 (1.13–1.37)**
	10–30 workers/day	**1.19 (1.15–1.22)**		**1.13 (1.09–1.17)**		**1.23 (1.17–1.29)**		**1.30 (1.18–1.43)**
	>30 workers/day	**1.30 (1.26–1.34)**		**1.28 (1.24–1.33)**		**1.24 (1.18–1.30)**		**1.47 (1.35–1.62)**
Nature of contacts								
	Working at home/alone	ref		ref		ref		ref
	Co-workers only	**1.36 (1.32–1.39)**		**1.30 (1.26–1.35)**		**1.43 (1.37–1.50)**		**1.28 (1.17–1.41)**
	General public	**1.18 (1.15–1.21)**		**1.12 (1.08–1.16)**		**1.18 (1.13–1.23)**		**1.39 (1.28–1.51)**
	Patients (with Covid-19)	**1.27 (1.22–1.33)**		**1.41 (1.34–1.50)**		1.01 (0.93–1.11)		**1.19 (1.01–1.39)**
Contaminated workspace								
	Homeworking/lone working	ref		ref		ref		ref
	Frequently sharing contact surfaces w/ co-workers	**1.34 (1.30–1.37)**		**1.29 (1.24–1.34)**		**1.40 (1.34–1.46)**		**1.28 (1.17–1.40)**
	Occasionally sharing contact surfaces w/ general public	**1.17 (1.14–1.21)**		**1.11 (1.06–1.15)**		**1.21 (1.15–1.27)**		**1.36 (1.23–1.51)**
	Frequently sharing contact surfaces w/ general public	**1.38 (1.35–1.42)**		**1.37 (1.32–1.42)**		**1.33 (1.27–1.40)**		**1.52 (1.39–1.66)**
Location								
	Working at home/alone	ref		ref		ref		ref
	Mostly outdoors	**1.32 (1.26–1.39)**		**1.28 (1.20–1.37)**		**1.36 (1.26–1.48)**		**1.32 (1.12–1.56)**
	Partly indoor	**1.33 (1.28–1.39)**		**1.26 (1.20–1.33)**		**1.45 (1.37–1.54)**		**1.19 (1.04–1.37)**
	Mostly indoor	**1.24 (1.21–1.27)**		**1.20 (1.17–1.24)**		**1.23 (1.18–1.28)**		**1.36 (1.26–1.47)**
Social distancing								
	Working at home/alone	ref		ref		ref		ref
	Always maintained	**1.21 (1.18–1.25)**		**1.18 (1.14–1.23)**		**1.26 (1.20–1.32)**		**1.16 (1.05–1.28)**
	Cannot always be maintained	**1.28 (1.25–1.31)**		**1.20 (1.16–1.24)**		**1.33 (1.28–1.39)**		**1.47 (1.35–1.60)**
	Can never be maintained	**1.26 (1.22–1.31)**		**1.35 (1.29–1.41)**		**1.09 (1.02–1.17)**		**1.25 (1.11–1.40)**
Face covering								
	Working at home/alone	ref		ref		ref		ref
	Always	**1.16 (1.13–1.19)**		**1.14 (1.11–1.18)**		**1.14 (1.10–1.19)**		**1.30 (1.20–1.40)**
	Not always while in proximity to others	**1.45 (1.41–1.49)**		**1.37 (1.32–1.42)**		**1.54 (1.47–1.61)**		**1.40 (1.27–1.53)**
	Not feasible	-		-		-		-
Income insecurity (%)								
	<1	ref		ref		ref		ref
	1–10	**1.15 (1.12–1.19)**		1.04 (0.99–1.08)		**1.26 (1.20–1.33)**		**1.34 (1.22–1.48)**
	11–25	**1.22 (1.15–1.30)**		**1.11 (1.02–1.21)**		**1.21 (1.09–1.34)**		**1.77 (1.51–2.08)**
	>25	**1.46 (1.41–1.51)**		**1.35 (1.29–1.42)**		**1.47 (1.39–1.56)**		**1.77 (1.61–1.96)**
Migrant workers (%)								
	<1	ref		ref		ref		ref
	1–10	**1.17 (1.12–1.23)**		**1.18 (1.11–1.26)**		**1.14 (1.05–1.23)**		1.20 (1.00–1.43)
	11–25	**1.35 (1.28–1.42)**		**1.31 (1.22–1.40)**		**1.36 (1.25–1.48)**		**1.41 (1.17–1.69)**
	>25	**1.65 (1.55–1.76)**		**1.59 (1.46–1.73)**		**1.76 (1.58–1.95)**		**1.50 (1.18–1.90)**
Residence area								
Province								
	Groningen	ref		ref		ref		ref
	Drenthe	**1.19 (1.08–1.31)**		**1.23 (1.08–1.39)**		**1.22 (1.04–1.43)**		0.95 (0.69–1.32)
	Flevoland	**1.54 (1.39–1.69)**		**1.79 (1.57–2.03)**		**1.28 (1.08–1.53)**		1.24 (0.90–1.71)
	Friesland	**1.36 (1.24–1.49)**		**1.26 (1.11–1.43)**		**1.49 (1.29–1.73)**		**1.42 (1.08–1.87)**
	Gelderland	**1.47 (1.37–1.58)**		**1.52 (1.38–1.68)**		**1.46 (1.29–1.65)**		**1.34 (1.07–1.67)**
	Limburg	**1.78 (1.65–1.93)**		**1.77 (1.60–1.97)**		**1.88 (1.65–2.13)**		**1.61 (1.26–2.05)**
	Noord-Brabant	**1.67 (1.55–1.80)**		**1.72 (1.56–1.90)**		**1.66 (1.47–1.88)**		**1.49 (1.20–1.85)**
	Noord-Holland	**1.59 (1.48–1.72)**		**1.59 (1.44–1.76)**		**1.59 (1.41–1.80)**		**1.75 (1.41–2.17)**
	Overijssel	**1.48 (1.37–1.60)**		**1.65 (1.48–1.83)**		**1.30 (1.13–1.49)**		**1.33 (1.05–1.69)**
	Utrecht	**1.25 (1.15–1.35)**		**1.37 (1.23–1.52)**		1.11 (0.97–1.27)		1.15 (0.91–1.45)
	Zeeland	**1.50 (1.36–1.65)**		**1.30 (1.13–1.50)**		**1.82 (1.55–2.13)**		**1.43 (1.04–1.96)**
	Zuid-Holland	**1.76 (1.64–1.89)**		**1.80 (1.64–1.99)**		**1.74 (1.54–1.96)**		**1.67 (1.35–2.07)**
Urbanity								
	Non-urban	ref		ref		ref		ref
	Mildly	1.02 (0.98–1.06)		**1.06 (1.00–1.12)**		0.96 (0.90–1.03)		1.04 (0.90–1.21)
	Moderate	1.01 (0.97–1.06)		**1.06 (1.00–1.13)**		0.95 (0.88–1.02)		1.03 (0.88–1.20)
	High	0.97 (0.93–1.01)		1.00 (0.94–1.05)		**0.91 (0.85–0.97)**		1.06 (0.92–1.23)
	Very high	**0.90 (0.86–0.94)**		0.95 (0.90–1.01)		**0.78 (0.73–0.84)**		1.07 (0.92–1.23)

^a^ All minimally adjusted analyses are adjusted for gender and age. ^b^ Wave 1: June 2020 to 8 February 2021, Wave 2: 9 February to 28 June 2021, Wave 3: 29 June to 31 August 2021.

A previous positive test decreased the odds of a positive test. Personal factors, such as low and intermediate educational levels, a non-western ethnic background, and being male, as well as living with older children increased the odds of a positive test (table 2). Significant differences in risk of infection between provinces were observed, with the highest infection rates generally found in the Southern provinces of The Netherlands (eg, Limburg, Brabant, Zuid-Holland). A very high urban area decreased the odds of a positive test for the entire study period, while no clear pattern between urbanity and the odds of a positive test was found (table 2).

After adjustment for a previous positive test, personal factors, household compositions and residence, for most of the dimensions of the COVID-19 JEM (eg, working at home/alone) increased odds for positive test were observed for the higher risk categories, compared to the lowest risk category (table 3, partially adjusted models). Over the entire study period, the odds for a positive test ranged from 1.03 (95% CI 1.00–1.07) for low income security to 1.13 for an elevated risk from contaminated workspaces (95% CI 1.09–1.16) and face covering (95% CI 1.10–1.16), and the highest proportion of workers with high job insecurity (95% CI 1.09–1.17). The partially adjusted odds showed similar patterns across the three pandemic waves, except for job insecurity and migrant workers. For high job insecurity, no significant increased odds for a positive test was observed at wave 1, in contrast to wave 2 and wave 3. A high risk due to the proportion of migrants was significant related to a positive test in wave 2 [OR 1.16 (95% CI 1.04–1.29)], but not for the other pandemic waves.

**Table 3:** Partially adjusted and fully adjusted odds ratios of occupational exposures with a positive COVID-19 test for the entire study period and for each pandemic wave ^c^. Reference (ref)=working at home/alone. **Bold indicates statistical significant (P<0.05)**.

		Total			Wave 1			Wave 2			Wave 3	
		OR (95%) ^a^	OR (95%) ^b^		OR (95%) ^a^	OR (95%) ^b^		OR (95%) ^a^	OR (95%) ^b^		OR (95%) ^a^	OR (95%) ^b^
Number of contacts (workers per day)												
	Home/alone	ref	*		ref	*		ref	*		ref	*
	<10	**1.08 (1.05–1.11)**			**1.07 (1.03–1.11)**			**1.10 (1.05–1.15)**			1.07 (0.97–1.18)	
	10–30	**1.06 (1.03–1.09)**			1.03 (0.99–1.07)			**1.07 (1.02–1.13)**			**1.11 (1.01–1.23)**	
	>30	**1.11 (1.08–1.15)**			**1.13 (1.09–1.18)**			1.04 (0.99–1.09)			**1.20 (1.09–1.33)**	
Nature of contacts												
	Home/alone	ref	ref		ref	ref		ref	ref		ref	ref
	Co-workers	**1.10 (1.07–1.13)**	0.97 (0.76–1.23)		**1.08 (1.04–1.12)**	1.02 (0.75–1.39)		**1.13 (1.08–1.18)**	0.85 (0.56–1.29)		1.05 (0.95–1.16)	1.17 (0.50–2.70)
	General public	**1.07 (1.05–1.10)**	0.91 (0.72–1.16)		**1.05 (1.02–1.09)**	0.99 (0.72–1.35)		**1.06 (1.01–1.11)**	0.74 (0.49–1.13)		**1.19 (1.09–1.29)**	1.17 (0.51–2.72)
	Patients (with COVID-19)	**1.09 (1.04–1.14)**	0.96 (0.75–1.23)		**1.23 (1.16–1.31)**	1.11 (0.80–1.54)		**0.85 (0.78–0.93)**	0.70 (0.45–1.08)		1.00 (0.85–1.18)	1.10 (0.46–2.62)
Contaminated workspaces (sharing contact surfaces)												
	Home/alone	ref	ref		ref	ref		ref	ref		ref	ref
	Frequently w/ co-workers	**1.10 (1.07–1.14)**	**1.26 (1.15–1.38)**		**1.09 (1.05–1.13)**	**1.25 (1.1–1.41)**		**1.12 (1.07–1.18)**	**1.25 (1.07–1.47)**		1.07 (0.97–1.18)	1.31 (0.96–1.79)
	Occasionally w/ general public	**1.09 (1.06–1.13)**	**1.31 (1.20–1.43)**		**1.06 (1.02–1.10)**	**1.26 (1.12–1.42)**		**1.11 (1.05–1.17)**	**1.40 (1.20–1.63)**		**1.17 (1.06–1.30)**	1.28 (0.95–1.71)
	Frequently w/ general public	**1.13 (1.09–1.16)**	**1.32 (1.20–1.44)**		**1.15 (1.10–1.19)**	**1.27 (1.12–1.43)**		**1.06 (1.00–1.11)**	**1.44 (1.23–1.69)**		**1.21 (1.10–1.34)**	1.21 (0.90–1.64)
Location												
	Home/alone	ref	ref		ref	ref		ref	ref		ref	ref
	Mostly outdoors	**1.10 (1.04–1.15)**	0.88 (0.73–1.05)		**1.08 (1.01–1.16)**	0.88 (0.69–1.11)		**1.11 (1.02–1.21)**	0.89 (0.65–1.22)		1.07 (0.90–1.27)	0.90 (0.46–1.76)
	Partly indoor	**1.07 (1.03–1.11)**	0.83 (0.69–1.01)		1.04 (0.98–1.09)	0.82 (0.64–1.05)		**1.13 (1.06–1.21)**	0.85 (0.61–1.19)		0.98 (0.85–1.14)	0.89 (0.44–1.82)
	Mostly indoor	**1.08 (1.06–1.11)**	0.84 (0.70–1.02)		**1.08 (1.04–1.11)**	0.85 (0.66–1.09)		**1.06 (1.02–1.10)**	0.83 (0.59–1.15)		**1.15 (1.06–1.25)**	0.97 (0.48–1.96)
Social distancing												
	Home/alone	ref	ref		ref	ref		ref	ref		ref	ref
	Always maintained	**1.06 (1.03–1.09)**	**0.56 (0.38–0.83)**		**1.05 (1.01–1.09)**	**0.55 (0.33–0.93)**		**1.08 (1.03–1.13)**	0.68 (0.35–1.31)		1.03 (0.93–1.14)	0.25 (0.06–1.04)
	Cannot always be maintained	**1.10 (1.07–1.13)**	**0.56 (0.38–0.83)**		**1.06 (1.03–1.10)**	**0.55 (0.32–0.92)**		**1.12 (1.07–1.17)**	0.68 (0.35–1.31)		**1.21 (1.11–1.32)**	0.26 (0.06–1.12)
	Can never be maintained	**1.08 (1.04–1.12)**	**0.56 (0.37–0.83)**		**1.18 (1.12–1.24)**	**0.58 (0.34–0.98)**		**0.91 (0.85–0.97)**	0.59 (0.30–1.14)		1.06 (0.94–1.19)	0.27 (0.06–1.16)
Face covering												
	Home/alone	ref	ref		ref	ref		ref	ref		ref	ref
	Always	**1.07 (1.04–1.09)**	**1.88 (1.31–2.70)**		**1.07 (1.03–1.10)**	**1.80 (1.11–2.91)**		1.04 (0.99–1.08)	1.80 (0.98–3.28)		**1.14 (1.05–1.24)**	3.00 (0.77–11.74)
	Not always while in proximity to others	**1.13 (1.10–1.16)**	**1.96 (1.36–2.82)**		**1.10 (1.06–1.15)**	**1.87 (1.15–3.03)**		**1.17 (1.12–1.23)**	**1.93 (1.05–3.53)**		1.08 (0.98–1.20)	2.79 (0.71–10.92)
	Not feasible	NA	NA		NA	NA		NA	NA		NA	NA
Income insecurity (%)												
	<1	ref	ref		ref	ref		ref	ref		ref	ref
	1–10	**1.03 (1.00–1.07)**	**1.04 (1.00–1.08)**		**0.95 (0.91–0.99)**	0.96 (0.91–1.01)		**1.13 (1.07–1.19)**	**1.12 (1.06–1.19)**		**1.16 (1.05–1.28)**	**1.14 (1.02–1.28)**
	11–25	1.04 (0.98–1.10)	1.00 (0.94–1.07)		0.97 (0.89–1.05)	0.96 (0.88–1.05)		1.02 (0.92–1.14)	0.94 (0.84–1.06)		**1.45 (1.23–1.70)**	**1.42 (1.18–1.71)**
	>25	**1.13 (1.09–1.17)**	**1.11 (1.05–1.17)**		**1.07 (1.02–1.13)**	**1.09 (1.01–1.17)**		**1.12 (1.05–1.19)**	1.05 (0.96–1.15)		**1.37 (1.23–1.52)**	**1.31 (1.12–1.54)**
Migrant workers (%)												
	<1	ref	ref		ref	ref		ref	ref		ref	ref
	1–10	0.98 (0.93–1.03)	0.99 (0.94–1.04)		1.01 (0.94–1.07)	1.00 (0.94–1.08)		0.95 (0.88–1.03)	0.97 (0.89–1.06)		0.96 (0.80–1.15)	0.98 (0.81–1.18)
	11–25	1.02 (0.97–1.08)	1.01 (0.96–1.07)		1.01 (0.95–1.09)	1.02 (0.95–1.10)		1.03 (0.94–1.12)	1.00 (0.92–1.10)		1.04 (0.86–1.25)	1.02 (0.84–1.25)
	>25	**1.10 (1.03–1.18)**	1.06 (0.98–1.14)		1.09 (0.99–1.19)	1.06 (0.96–1.17)		**1.16 (1.04–1.29)**	1.08 (0.96–1.22)		1.04 (0.81–1.32)	1.02 (0.78–1.32)

^a^ Partially adjusted analyses are corrected for a previous positive test, all personal characteristics and residence area (supplementary file 3). ^b^ Fully analyses are corrected for a previous positive test, personal characteristics, residence area and occupational exposure (supplementary file 3). ^c^ Wave 1: June 2020 to 8 February 2021, Wave 2: 9 February to 28 June 2021, Wave 3: 29 June to 31 August 2021. * Due to collinearity, number of contacts was not included in the fully adjusted models.

In the fully adjusted multiple regression models, all models were adjusted for a previous positive test, personal factors, household composition, residence area and all other dimensions of occupational exposure (table 3). We had to exclude number of contacts as a risk factor because of multicollinearity with nature of contacts and contaminated workspaces (correlation >0.9, supplementary table S2). A higher risk of exposure due to contaminated work spaces or low levels of use of face coverings resulted in higher odds of a positive test for the entire study period [OR 1.32 (95% CI 1.20–1.44) and OR 1.96 (95% CI 1.36–2.82), respectively] and across the first two pandemic waves [OR 1.27 (95% CI 1.12–1.43) and OR 1.87 (95% CI 1.15–3.03)]. Exposure to income insecurity resulted in higher odds of a positive test in the entire study period [up to: OR 1.11 (95% CI 1.05-1.17)) and in wave 1 (up to: OR 1.09 (95% CI 1.01–1.17)] and wave 3 [up to: OR 1.31 (95% CI 1.12–1.54)]. After adjusting for all other factors, exposure risks due to lack of social distance actually resulted in lower odds of a positive test in the overall period [OR 0.56 (95% CI 0.37–0.83)] and wave 1 [OR 0.58 (95% CI 0.34–0.98)]. The associations disappeared for nature of contacts, location and migrants workers for the entire study period and all three pandemic waves. Supplementary table S3 shows the OR and 95% CI for all variables included in the partially and fully adjusted models.

**Figure 1:**
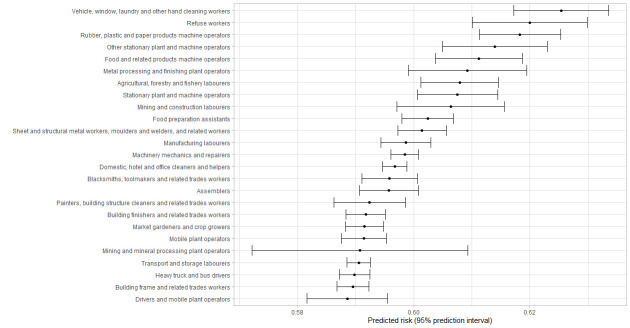
First 25 jobs with the highest predicted risks for a positive test for the total period, predicted risks are expressed as predicted probabilities.

**Figure 2:**
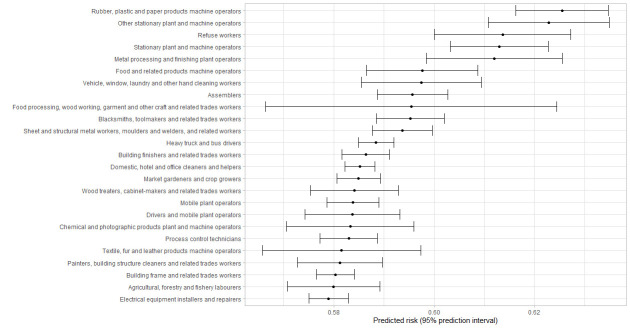
First 25 jobs with the highest predicted values for a positive tests for Wave 1, predicted risks are expressed as predicted probabilities.

**Figure 3:**
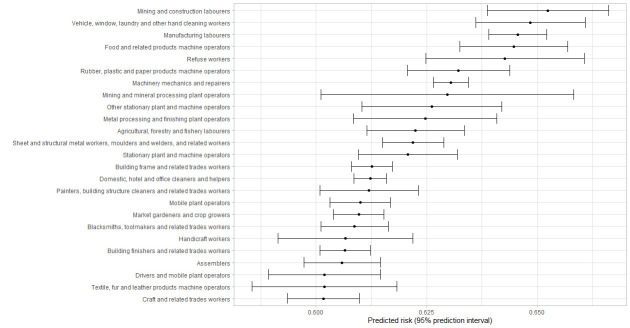
First 25 jobs with the highest predicted values for a positive tests for Wave 2, predicted risks are expressed as predicted probabilities.

**Figure 4:**
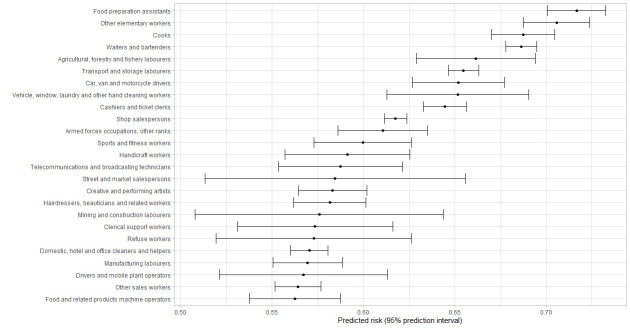
First 25 jobs with the highest predicted values for a positive tests for Wave 3, predicted risks are expressed as predicted probabilities.

Figure 1–4 shows the 25 jobs with the highest predicted risk of a positive test for the entire pandemic period and across the three different pandemic waves. Regarding the entire study period, cleaners, refuse workers and machine operators had the highest predictive risk of a positive test. The three pandemic waves showed differences, refuse workers and machine operators have the highest risk in wave 1, mining and construction labors, cleaning workers and machine operators in wave 2, and food preparation assistants, cooks, other elementary workers, waiters and bartenders in wave 3.

## Discussion

Nine percent of tests were positive during the entire study period and being exposed at work was related to a higher risk of a positive test in the period July 2020 to August 2021. The fully adjusted models suggest that contaminated workspaces, (lack of) face covering and income insecurity are the most important work-related risk factors for a positive test. Some differences were found over the study pandemic waves; contaminated workspaces and face covering were relevant in the first two pandemic waves, whereas income insecurity was especially important in the third wave. The risk to have a positive test differed across occupations and over time, whereby cleaning workers, refuse workers and machine operators had the highest predicted risk of a positive COVID-19 test for the entire pandemic period.

The minimally adjusted analyses showed that occupational exposures are associated with a positive test, which is in line with previous research on occupational exposure and COVID-19 infections (31–33). After correcting for other factors including children living at home and residence area, occupational exposure to Sars-Cov-2 still elevated the risk to be infected but became smaller for all dimensions and across all pandemic waves. Similarly, Rhodes et al (33) showed that the clear exposure–response relationship for the COVID-19 JEM dimensions on transmission risk and mitigation measures were reduced and even disappeared over time (33). The reason why the current study did not observe a complete disappearance could be explained by that fact that the current study covered a shorter period with still many restrictions and no roll out of the booster vaccination program yet.

After adjusting for all dimensions, contaminated workspaces, social distancing, face covering and job insecurity remained significantly associated with a higher risk of a positive test. For income insecurity, the odds for a positive test was highest in wave 3 (June–August 2021), probably due to the relaxation of measures whereby workers in occupations with a high income insecurity (eg, restaurant, retailers) were allowed to work. Due to collinearity between the dimensions, we could not draw any conclusion on which specific occupational exposure was the most important to drive the observed differences in infections. For example, even though it is unknown to what extent surface contamination contribute to outbreaks, the current study showed clear associations with a positive test. It might be that surface contamination also covers social distancing and nature of contacts. Furthermore, scientific evidence has shown that airborne transmission more often occurs in enclosed environments with poor ventilation (34), and poor ventilation is thereby also a risk factor in the work setting (35). The COVID-19-JEM did not include exposure to poor ventilated workplaces. Future research on occupational exposure and COVID-19 is needed to investigate the importance of each transmission risk including ventilation and vaccinations, which ask for an updated version of the COVID-19-JEM.

The risk to be infected differed across occupations and over time, with the highest predicted risk of a positive COVID-19 test for cleaning workers, refuse workers and machine operators over the entire period coved in this study. These results are in line with other studies identifying groups with a higher risk of a positive COVID-19 test (36), hospital admission (5, 37, 38) or even mortality (9–11). Occupations with the highest predictive risk varied over time, with cleaners, refuse workers and operators at the top during the first wave, operators and other high skilled workers during the second wave and food preparation assistants, waiters, bartenders, and cooks in the last wave. The latter is not surprising due to the reopening of restaurants and bars. Contrary to previous research (10, 11, 33, 38), it should be noticed that healthcare workers were not amongst the group of occupations with the highest risk in the current study. This can be explained by the absence of data of the first months of the COVID-19 pandemic in The Netherlands and the absence of test data from test centers located within hospitals. This probably resulted in an underestimation of the risks for infection at work in healthcare workers.

Strengths of the current study include its large study population, administrative data on all variables, except occupation, and distinguishing three different waves. Another major strength was the use of the test-negative design to avoid selection bias, where participants with a positive test were compared to those who were tested negative (27). As noted above, the test-negative design is intended to address biases arising from differences in the propensity to be tested. Whether someone was actually tested was a personal choice, influenced by symptoms, availability of healthcare, health seeking behavior, etc. This is exactly the situation that the test-negative design is intended to address, since it provides adjustment for these personal differences in propensity to be tested (22). However, two potential limitations of the test-negative design should be acknowledged. Firstly, the test-negative design will not provide valid effect estimates for factors that also affect the risks of other infections (22); we consider it unlikely that the occupations we have considered have increased risks for other infections during the pandemic period of the current study, but of course this cannot be ruled out. Secondly, it would have been desirable to also adjust for the reason for testing (39), but unfortunately this information was not available. This would be likely to lead to an underestimation of the OR for occupations which are regularly tested, in comparison with occupations that only involve symptomatic testing. Once again, we do not consider that the main occupations we have focused on would have substantial differences in testing policy, but this cannot be ruled out. In this context, we note that for almost all of the variables considered, we have found similar findings across the three waves, when different testing policies were operating; this would not be the case if the above-mentioned biases were having a significant effect on our findings. Some other limitations should be noted as well. Firstly, the sample size in the current study is not representative for the general working population as it does not include self-employed people and underrepresent workers that just entered the labor force. In addition, a relatively large number of workers were excluded from the dataset as they left paid employment between their DLFS. Due to selection within the DLFS, workers were more often higher educated (46%), with a Dutch background (86%) and female (51%) than in the Dutch working population (42%, 75% and 47%, respectively) (40). Moreover, it is likely that certain occupations will be underrepresented in the DFLS survey, such as those with a high proportion of low educated workers or non-white ethnic workers. Secondly, the CoronaIT database on Sars-Cov-2 infections has several limitations such as the time period (eg, missing the first months of the pandemic and no access from August 2021 onwards) and the absence of test results of some test centers (ie, hospitals and commercial test centers). Thirdly, as no information is available on the vaccination status of workers, we were not able to take this into account as variable which might differ across occupations (41). Fourth, the COVID-19-JEM was developed for a basic state, meaning that all sectors are (partly) open. However, different governmental measures were announced for specific months, meaning that some sectors were closed for a specific period, while the JEM indicate the jobs in these sectors are at elevated or high risk. As measures quickly changed over time, we were not able to adjust the JEM to all different measures in the current study.

### Concluding remarks

Occupational exposures are associated with a higher risk of a positive COVID-19 test among workers. Even though adjusting for other factors strongly reduced the odds of being infected, most occupational dimensions remained at elevated risk. The multiple regression models showed that contaminated workspaces, (lack of) face covering and income insecurity are the most important risk factors for a positive test. Some occupations were at higher risk of being infected at the workplace than others, and variations in occupations with the highest risk exist over time. These findings provide insights for interventions among workers for future pandemic waves of COVID-19 or other respiratory epidemics.

## Supplementary material

Supplementary material

## Data Availability

Data are stored at Statistics Netherlands. Data are available upon reasonable request, following the guidelines of Statistics Netherlands.
