# Supplementary material for: The impact of occupational exposures on infection rates during the COVID-19 pandemic: a test-negative design study with register data of 207 034 Dutch workers
Source: Scand J Work Environ Health. 2023 May 1;49(4):259–70. doi: 10.5271/sjweh.4086 (PMC10713988; doi:10.5271/sjweh.4086)
Supplement: Supplementary material [file SJWEH-49-259-S001.pdf]

The impact of occupational exposures on infection rates during the COVID-19 pandemic: a test-negative design study with register data of 207 034 Dutch workers[1]

by Iris Eekhout, PhD,[2] Martie van Tongeren, PhD, Neil Pearce, PhD, Karen M Oude Hengel, PhD

1. *Supplementary material*
2. *Correspondence to: Karen M. Oude Hengel, Netherlands Organisation for Applied Scientific Research TNO, Unit Healthy Living, Sylviusweg 71, 2333 BE Leiden, The Netherlands. [E-mail: karen.oudehengel@tno.nl]*

Table S1. JEM scores of the study population for the entire study period and for each pandemic wave[1]

**Transmission risk factors**	**Total** N(%)	**Wave 1** N(%)	**Wave 2** N(%)	**Wave 3** N(%)
Number of contact				
- No risk	68198(32.9)	48278(32.6)	35074(33.1)	12030(34.8)
- Low risk	51129(24.7)	35647(24.1)	25814(24.3)	7595(22)
- Elevated risk	43907(21.2)	32377(21.9)	23099(21.8)	7112(20.6)
- High risk	43800(21.2)	31667(21.4)	22095(20.8)	7864(22.7)
Nature of contacts				
- No risk	68196(32.9)	48276(32.6)	35072(33.1)	12030(34.8)
- Low risk	50296(24.3)	34495(23.3)	25032(23.6)	7318(21.1)
- Elevated risk	75880(36.7)	56022(37.9)	40010(37.7)	12937(37.4)
- High risk	12662(6.1)	9176(6.2)	5968(5.6)	2316(6.7)
Contaminated workspaces				
- No risk	72590(35.1)	51624(34.9)	37573(35.4)	12922(37.3)
- Low risk	52463(25.3)	36322(24.5)	26406(24.9)	7696(22.2)
- Elevated risk	33137(164.0)	24979(16.9)	17653(16.6)	5286(15.3)
- High risk	48844(23.6)	35044(23.7)	24450(234.0)	8697(25.1)
Location				
- No risk	67471(32.6)	47767(32.3)	34753(32.8)	11925(34.5)
- Low risk	8676(4.2)	5941(4)	4216(4.0)	1337(3.9)
- Elevated risk	16058(7.8)	11102(7.5)	8194(7.7)	2286(6.6)
- High risk	114829(55.5)	83159(56.2)	58919(55.5)	19053(55.1)
**Mitigation factors**				
Social distancing				
- No risk	69100(33.4)	48888(334.0)	35537(33.5)	12179(35.2)
- Low risk	48559(23.5)	34123(23.1)	24560(23.2)	7442(21.5)
- Elevated risk	66387(32.1)	48521(32.8)	34546(32.6)	10467(30.3)
- High risk	22988(11.1)	16437(11.1)	11439(10.8)	4513(134.0)
Face covering				
- No risk	69313(33.5)	49039(33.1)	35645(33.6)	12220(35.3)
- Low risk	96123(46.4)	70302(47.5)	49517(46.7)	16217(46.9)
- Elevated risk	41598(20.1)	28628(19.3)	20920(19.7)	6164(17.8)
- High risk				
**Precarious work**				
Income insecurity				
- No risk	162024(78.3)	116188(78.5)	83860(79.1)	26911(77.8)
- Low risk	22540(10.9)	16042(10.8)	11444(10.8)	3832(11.1)
- Elevated risk	5514(2.7)	3878(2.6)	2605(2.5)	916(2.6)
- High risk	16956(8.2)	11861(8)	8173(7.7)	2942(8.5)
Migrant workers				
- No risk	10158(4.9)	7763(5.2)	5881(5.5)	1645(4.8)
- Low risk	132137(63.8)	95092(64.3)	68275(64.4)	22615(65.4)
- Elevated risk	55794(26.9)	38998(26.4)	27596(26)	9037(26.1)
- High risk	8945(4.3)	6116(4.1)	4330(4.1)	1304(3.8)

[1] Wave 1: June 2020 to February 8 2021, Wave 2: February 9 2021 to June 28 2021, Wave 3 June 29 2021 to August 31 2021

Table S2. Correlation between JEM scores for the entire study period

	Number of contacts	Nature of contacts	Contaminated workspaces	Location	Social distance	Face covering	Income insecurity	Migrants
Number of contacts	1.00							
Nature of contacts	0.90	1.00						
Contaminated workspaces	0.93	0.88	1.00					
Location	0.82	0.88	0.78	1.00				
Social distance	0.89	0.91	0.89	0.83	1.00			
Face covering	0.58	0.62	0.58	0.78	0.69	1.00		
Income insecurity	0.37	0.27	0.41	0.27	0.23	0.24	1.00	
Migrants	-0.15	-0.17	-0.11	-0.05	-0.14	0.15	0.24	1.00

Table S3. Partially adjusted[1] and fully adjusted[2] odds ratios for occupational exposures and other factors with a positive COVID-19 test for entire study period and for each pandemic wave[3]

	**Total**		**Wave 1**		**Wave 2**		**Wave 3**	
	Partially adjusted OR (95%)	Fully adjusted OR (95%)	Partially adjusted OR (95%)	Fully adjusted OR (95%)	Partially adjusted OR (95%)	Fully adjusted OR (95%)	Partially adjusted OR (95%)	Fully adjusted OR (95%)
**Number of contacts**								
Working at home/alone	ref	ref	ref	ref	ref	ref	ref	ref
<10 workers/day	**1.08 (1.05 - 1.11)**		**1.07 (1.03 - 1.11)**		**1.10 (1.05 - 1.15)**		1.07 (0.97 - 1.18)	
10-30 workers/day	**1.06 (1.03 - 1.09)**		1.03 (0.99 - 1.07)		**1.07 (1.02 - 1.13)**		**1.11 (1.01 - 1.23)**	
>30 workers/day	**1.11 (1.08 - 1.15)**		**1.13 (1.09 - 1.18)**		1.04 (0.99 - 1.09)		**1.20 (1.09 - 1.33)**	
**Nature of contacts**								
Working at home/alone	ref	ref	ref	ref	ref	ref	ref	ref
co-workers only	**1.10 (1.07 - 1.13)**	0.97 (0.76 - 1.23)	**1.08 (1.04 - 1.12)**	1.02 (0.75 - 1.39)	**1.13 (1.08 - 1.18)**	0.85 (0.56 - 1.29)	1.05 (0.95 - 1.16)	1.17 (0.50 - 2.70)
General public	**1.07 (1.05 - 1.10)**	0.91 (0.72 - 1.16)	**1.05 (1.02 - 1.09)**	0.99 (0.72 - 1.35)	**1.06 (1.01 - 1.11)**	0.74 (0.49 - 1.13)	**1.19 (1.09 - 1.29)**	1.17 (0.51 - 2.72)
Patients (with Covid-19)	**1.09 (1.04 - 1.14)**	0.96 (0.75 - 1.23)	**1.23 (1.16 - 1.31)**	1.11 (0.80 - 1.54)	**0.85 (0.78 - 0.93)**	0.70 (0.45 - 1.08)	1.00 (0.85 - 1.18)	1.10 (0.46 - 2.62)
**Contaminated workspaces**								
Homeworking/lone working	ref	ref	ref	ref	ref	ref	ref	ref
Frequently sharing contact surfaces with co-workers	**1.10 (1.07 - 1.14)**	**1.26 (1.15 - 1.38)**	**1.09 (1.05 - 1.13)**	**1.25 (1.1 - 1.41)**	**1.12 (1.07 - 1.18)**	**1.25 (1.07 - 1.47)**	1.07 (0.97 - 1.18)	1.31 (0.96 - 1.79)
Occasionally sharing contact surfaces with general public	**1.09 (1.06 - 1.13)**	**1.31 (1.20 - 1.43)**	**1.06 (1.02 - 1.10)**	**1.26 (1.12 - 1.42)**	**1.11 (1.05 - 1.17)**	**1.40 (1.20 - 1.63)**	**1.17 (1.06 - 1.30)**	1.28 (0.95 - 1.71)
Frequently sharing contact surfaces with general public	**1.13 (1.09 - 1.16)**	**1.32 (1.20 - 1.44)**	**1.15 (1.10 - 1.19)**	**1.27 (1.12 - 1.43)**	**1.06 (1.00 - 1.11)**	**1.44 (1.23 - 1.69)**	**1.21 (1.10 - 1.34)**	1.21 (0.9 - 1.64)
**Location**								
Working at home/alone	ref	ref	ref	ref	ref	ref	ref	ref
Mostly outdoors	**1.10 (1.04 - 1.15)**	0.88 (0.73 - 1.05)	**1.08 (1.01 - 1.16)**	0.88 (0.69 - 1.11)	**1.11 (1.02 - 1.21)**	0.89 (0.65 - 1.22)	1.07 (0.90 - 1.27)	0.90 (0.46 - 1.76)
Partly indoor	**1.07 (1.03 - 1.11)**	0.83 (0.69 - 1.01)	1.04 (0.98 - 1.09)	0.82 (0.64 - 1.05)	**1.13 (1.06 - 1.21)**	0.85 (0.61 - 1.19)	0.98 (0.85 - 1.14)	0.89 (0.44 - 1.82)
Mostly indoor	**1.08 (1.06 - 1.11)**	0.84 (0.70 - 1.02)	**1.08 (1.04 - 1.11)**	0.85 (0.66 - 1.09)	**1.06 (1.02 - 1.10)**	0.83 (0.59 - 1.15)	**1.15 (1.06 - 1.25)**	0.97 (0.48 - 1.96)
**Social distancing**								
Working at home/alone	ref	ref	ref	ref	ref	ref	ref	ref

Always maintained	**1.06 (1.03 - 1.09)**	**0.56 (0.38 - 0.83)**	**1.05 (1.01 - 1.09)**	**0.55 (0.33 - 0.93)**	**1.08 (1.03 - 1.13)**	0.68 (0.35 - 1.31)	1.03 (0.93 - 1.14)	0.25 (0.06 - 1.04)
Cannot always be maintained	**1.10 (1.07 - 1.13)**	**0.56 (0.38 - 0.83)**	**1.06 (1.03 - 1.10)**	**0.55 (0.32 - 0.92)**	**1.12 (1.07 - 1.17)**	0.68 (0.35 - 1.31)	**1.21 (1.11 - 1.32)**	0.26 (0.06 - 1.12)
Can never be maintained	**1.08 (1.04 - 1.12)**	**0.56 (0.37 - 0.83)**	**1.18 (1.12 - 1.24)**	**0.58 (0.34 - 0.98)**	**0.91 (0.85 - 0.97)**	0.59 (0.30 - 1.14)	1.06 (0.94 - 1.19)	0.27 (0.06 - 1.16)
**Face covering**								
Working at home/alone	ref	ref	ref	ref	ref	ref	ref	ref
Always	**1.07 (1.04 - 1.09)**	**1.88 (1.31 - 2.70)**	**1.07 (1.03 - 1.10)**	**1.80 (1.11 - 2.91)**	1.04 (0.99 - 1.08)	1.80 (0.98 - 3.28)	**1.14 (1.05 - 1.24)**	3.00 (0.77 - 11.74)
Not always while in proximity to others	**1.13 (1.10 - 1.16)**	**1.96 (1.36 - 2.82)**	**1.10 (1.06 - 1.15)**	**1.87 (1.15 - 3.03)**	**1.17 (1.12 - 1.23)**	**1.93 (1.05 - 3.53)**	1.08 (0.98 - 1.20)	2.79 (0.71 - 10.92)
Not feasible	-	-	-	-	-	-	-	-
**Income insecurity**								
<1%	ref	ref	ref	ref	ref	ref	ref	ref
1-10%	**1.03 (1.00 - 1.07)**	**1.04 (1.00 - 1.08)**	**0.95 (0.91 - 0.99)**	0.96 (0.91 - 1.01)	**1.13 (1.07 - 1.19)**	**1.12 (1.06 - 1.19)**	**1.16 (1.05 - 1.28)**	**1.14 (1.02 - 1.28)**
11-25%	1.04 (0.98 - 1.10)	1.00 (0.94 - 1.07)	0.97 (0.89 - 1.05)	0.96 (0.88 - 1.05)	1.02 (0.92 - 1.14)	0.94 (0.84 - 1.06)	**1.45 (1.23 - 1.70)**	**1.42 (1.18 - 1.71)**
>25%	**1.13 (1.09 - 1.17)**	**1.11 (1.05 - 1.17)**	**1.07 (1.02 - 1.13)**	**1.09 (1.01 - 1.17)**	**1.12 (1.05 - 1.19)**	1.05 (0.96 - 1.15)	**1.37 (1.23 - 1.52)**	**1.31 (1.12 - 1.54)**
**Migrant workers**								
<1%	ref	ref	ref	ref	ref	ref	ref	ref
1-10%	0.98 (0.93 - 1.03)	0.99 (0.94 - 1.04)	1.01 (0.94 - 1.07)	1.00 (0.94 - 1.08)	0.95 (0.88 - 1.03)	0.97 (0.89 - 1.06)	0.96 (0.80 - 1.15)	0.98 (0.81 - 1.18)
11-25%	1.02 (0.97 - 1.08)	1.01 (0.96 - 1.07)	1.01 (0.95 - 1.09)	1.02 (0.95 - 1.10)	1.03 (0.94 - 1.12)	1.00 (0.92 - 1.10)	1.04 (0.86 - 1.25)	1.02 (0.84 - 1.25)
>25%	**1.10 (1.03 - 1.18)**	1.06 (0.98 - 1.14)	1.09 (0.99 - 1.19)	1.06 (0.96 - 1.17)	**1.16 (1.04 - 1.29)**	1.08 (0.96 - 1.22)	1.04 (0.81 - 1.32)	1.02 (0.78 - 1.32)
**Previous positive testresult**	**0.38 (0.35 - 0.42)**	**0.38 (0.34 - 0.41)**	**0.49 (0.40 - 0.61)**	**0.49 (0.40 - 0.61)**	**0.37 (0.33 - 0.42)**	**0.37 (0.33 - 0.42)**	**0.33 (0.27 - 0.40)**	**0.33 (0.27 - 0.40)**
**Age**	**1.00 (1.00 - 1.00)**	**1.00 (1.00 - 1.00)**	**1.01 (1.00 - 1.01)**	**1.01 (1.00 - 1.01)**	**1.00 (1.00 - 1.00)**	**1.00 (1.00 - 1.00)**	**0.98 (0.97 - 0.98)**	**0.98 (0.97 - 0.98)**
**Gender**								
Female	ref	ref	ref	ref	ref	ref	ref	ref
Male	**1.25 (1.22 - 1.27)**	**1.25 (1.22 - 1.28)**	**1.24 (1.21 - 1.28)**	**1.27 (1.23 - 1.31)**	**1.26 (1.22 - 1.30)**	**1.21 (1.16 - 1.25)**	**1.23 (1.15 - 1.31)**	**1.27 (1.18 - 1.36)**
**Educational level**								
Low	**1.61 (1.56 - 1.66)**	**1.48 (1.43 - 1.53)**	**1.54 (1.47 - 1.60)**	**1.45 (1.38 - 1.51)**	**1.72 (1.64 - 1.82)**	**1.55 (1.46 - 1.64)**	**1.41 (1.26 - 1.57)**	**1.28 (1.13 - 1.44)**
Intermediate	**1.38 (1.35 - 1.41)**	**1.31 (1.28 - 1.34)**	**1.35 (1.31 - 1.39)**	**1.29 (1.25 - 1.33)**	**1.41 (1.36 - 1.47)**	**1.34 (1.28 - 1.4)**	**1.38 (1.28 - 1.49)**	**1.30 (1.20 - 1.42)**
High	ref	ref	ref	ref	ref	ref	ref	ref
**Ethnic background**								
Dutch	ref	ref	ref	ref	ref	ref	ref	ref

Non-Western	**1.56 (1.50 - 1.62)**	**1.53 (1.48 - 1.59)**	**1.68 (1.60 - 1.77)**	**1.66 (1.58 - 1.74)**	**1.46 (1.37 - 1.55)**	**1.42 (1.33 - 1.52)**	**1.34 (1.20 - 1.50)**	**1.33 (1.18 - 1.49)**
Western	1.00 (0.96 - 1.04)	1.00 (0.96 - 1.04)	0.98 (0.93 - 1.04)	0.99 (0.93 - 1.04)	1.01 (0.94 - 1.08)	1.00 (0.93 - 1.07)	1.09 (0.96 - 1.24)	1.08 (0.95 - 1.23)
**Household position**								
Single household	ref	ref	ref	ref	ref	ref	ref	ref
Single parent	0.98 (0.92 - 1.04)	0.98 (0.92 - 1.05)	0.98 (0.90 - 1.07)	0.98 (0.90 - 1.07)	0.95 (0.86 - 1.06)	0.96 (0.86 - 1.07)	1.15 (0.94 - 1.41)	1.17 (0.95 - 1.43)
Part of a couple	**1.16 (1.12 - 1.21)**	**1.17 (1.12 - 1.22)**	**1.25 (1.18 - 1.32)**	**1.25 (1.19 - 1.32)**	**1.09 (1.02 - 1.17)**	**1.10 (1.02 - 1.17)**	1.00 (0.89 - 1.13)	1.02 (0.90 - 1.15)
Other	**1.18 (1.11 - 1.25)**	**1.15 (1.09 - 1.22)**	**1.20 (1.11 - 1.30)**	**1.18 (1.09 - 1.28)**	1.05 (0.96 - 1.16)	1.03 (0.94 - 1.14)	1.18 (0.99 - 1.39)	1.12 (0.95 - 1.33)
**Children living at home**								
None	ref	ref	ref	ref	ref	ref	ref	ref
Children below 12 years	**0.75 (0.73 - 0.78)**	**0.76 (0.74 - 0.79)**	**0.77 (0.74 - 0.81)**	**0.78 (0.74 - 0.81)**	**0.81 (0.76 - 0.85)**	**0.81 (0.77 - 0.86)**	**0.63 (0.56 - 0.71)**	**0.64 (0.57 - 0.72)**
Children above 12 years	**1.34 (1.30 - 1.37)**	**1.34 (1.30 - 1.37)**	**1.34 (1.30 - 1.39)**	**1.34 (1.30 - 1.39)**	**1.40 (1.34 - 1.47)**	**1.40 (1.34 - 1.47)**	**1.19 (1.07 - 1.32)**	**1.19 (1.07 - 1.32)**
Children in both age groups	**1.23 (1.18 - 1.28)**	**1.23 (1.18 - 1.28)**	**1.28 (1.22 - 1.35)**	**1.28 (1.22 - 1.36)**	**1.21 (1.13 - 1.30)**	**1.22 (1.14 - 1.31)**	1.14 (0.98 - 1.32)	1.14 (0.98 - 1.32)
**Province**								
Groningen	ref	ref	ref	ref	ref	ref	ref	ref
Drenthe	1.08 (0.98 - 1.19)	1.08 (0.98 - 1.19)	1.12 (0.98 - 1.27)	1.12 (0.98 - 1.27)	1.08 (0.92 - 1.26)	1.08 (0.92 - 1.27)	0.93 (0.67 - 1.29)	0.93 (0.67 - 1.29)
Flevoland	**1.33 (1.20 - 1.47)**	**1.34 (1.21 - 1.48)**	**1.54 (1.35 - 1.76)**	**1.56 (1.36 - 1.78)**	1.11 (0.93 - 1.32)	1.11 (0.93 - 1.32)	1.11 (0.8 - 1.54)	1.11 (0.80 - 1.55)
Friesland	**1.24 (1.13 - 1.35)**	**1.23 (1.13 - 1.35)**	**1.16 (1.02 - 1.32)**	**1.16 (1.02 - 1.32)**	**1.31 (1.13 - 1.52)**	**1.30 (1.12 - 1.51)**	**1.39 (1.05 - 1.84)**	**1.40 (1.06 - 1.86)**
Gelderland	**1.37 (1.27 - 1.48)**	**1.38 (1.28 - 1.48)**	**1.43 (1.29 - 1.58)**	**1.43 (1.29 - 1.58)**	**1.33 (1.17 - 1.51)**	**1.34 (1.18 - 1.52)**	**1.31 (1.04 - 1.65)**	**1.31 (1.04 - 1.64)**
Limburg	**1.64 (1.52 - 1.77)**	**1.63 (1.51 - 1.77)**	**1.64 (1.47 - 1.82)**	**1.63 (1.46 - 1.81)**	**1.70 (1.49 - 1.94)**	**1.70 (1.49 - 1.94)**	**1.53 (1.20 - 1.95)**	**1.52 (1.19 - 1.94)**
Noord-Brabant	**1.57 (1.46 - 1.69)**	**1.57 (1.46 - 1.69)**	**1.62 (1.46 - 1.79)**	**1.62 (1.47 - 1.79)**	**1.55 (1.37 - 1.75)**	**1.54 (1.36 - 1.75)**	**1.47 (1.18 - 1.83)**	**1.47 (1.18 - 1.84)**
Noord-Holland	**1.58 (1.46 - 1.70)**	**1.58 (1.47 - 1.70)**	**1.56 (1.41 - 1.73)**	**1.57 (1.42 - 1.74)**	**1.59 (1.40 - 1.80)**	**1.59 (1.40 - 1.80)**	**1.72 (1.38 - 2.15)**	**1.72 (1.38 - 2.15)**
Overijssel	**1.38 (1.27 - 1.49)**	**1.38 (1.27 - 1.49)**	**1.54 (1.38 - 1.72)**	**1.55 (1.39 - 1.72)**	**1.18 (1.02 - 1.35)**	**1.17 (1.02 - 1.35)**	**1.30 (1.02 - 1.67)**	**1.31 (1.02 - 1.67)**
Utrecht	**1.28 (1.19 - 1.39)**	**1.29 (1.20 - 1.40)**	**1.39 (1.25 - 1.55)**	**1.40 (1.26 - 1.56)**	**1.16 (1.01 - 1.33)**	**1.16 (1.02 - 1.33)**	1.23 (0.97 - 1.56)	1.24 (0.97 - 1.57)
Zeeland	**1.33 (1.20 - 1.47)**	**1.32 (1.20 - 1.46)**	**1.16 (1.01 - 1.34)**	**1.16 (1.01 - 1.34)**	**1.55 (1.32 - 1.82)**	**1.55 (1.32 - 1.82)**	1.39 (1.01 - 1.91)	**1.39 (1.01 - 1.92)**
Zuid-Holland	**1.72 (1.60 - 1.86)**	**1.73 (1.61 - 1.86)**	**1.75 (1.58 - 1.93)**	**1.76 (1.59 - 1.94)**	**1.74 (1.54 - 1.97)**	**1.74 (1.54 - 1.97)**	**1.63 (1.31 - 2.03)**	**1.65 (1.32 - 2.05)**
**Urbanity**								
Non-urban	ref	ref	ref	ref	ref	ref	ref	ref
Mildly	0.96 (0.92 – 1.00)	0.96 (0.92 – 1.00)	0.98 (0.92 - 1.03)	0.98 (0.92 - 1.03)	0.94 (0.87 – 1.00)	0.94 (0.87 - 1.01)	0.99 (0.85 - 1.16)	1.00 (0.85 - 1.17)
Moderate	**0.94 (0.90 - 0.99)**	**0.95 (0.90 - 0.99)**	0.96 (0.90 - 1.02)	0.96 (0.91 - 1.03)	**0.91 (0.85 - 0.98)**	**0.92 (0.85 - 0.99)**	0.96 (0.81 - 1.14)	0.96 (0.81 - 1.13)
High	**0.90 (0.86 - 0.94)**	**0.90 (0.86 - 0.94)**	**0.90 (0.84 - 0.95)**	**0.90 (0.85 - 0.96)**	**0.88 (0.82 - 0.94)**	**0.89 (0.83 - 0.95)**	0.99 (0.85 - 1.16)	0.98 (0.84 - 1.15)
Very High	**0.84 (0.8 0- 0.88)**	**0.84 (0.80 - 0.88)**	**0.86 (0.81 - 0.92)**	**0.87 (0.81 - 0.93)**	**0.74 (0.69 - 0.81)**	**0.75 (0.70 - 0.82)**	1.01 (0.85 - 1.19)	1.00 (0.84 - 1.18)

[1] Partially adjusted analyses are corrected for a previous positive test, all personal characteristics and residence area.; [2] Fully analyses are corrected for a previous positive test, all personal characteristics, residence area and occupational exposure.[2] Wave 1: June 2020 to February 8 2021, Wave 2: February 9 2021 to June 28 2021, Wave 3 June 29 2021 to August 31 2021. * Due to collinearity, number of contacts was not included in the fully adjusted models; Bold indicates statistical significant (P<0.05).
